# Male adolescents' (Aged 15-24 years) smoking habit and its determinant: analysis of Indonesia demographic and health survey data, 2017

**DOI:** 10.4314/ahs.v24i4.45

**Published:** 2024-12

**Authors:** Diana Rohmandani Putri, Erni Astutik, Putri Bungsu Machmud, Tika Dwi Tama

**Affiliations:** 1 Department of Epidemiology, Biostatistics, Population Studies, and Health Promotion, Faculty of Public Health, Universitas Airlangga, Surabaya, Indonesia; 2 Department of Epidemiology, Faculty of Public Health, Universitas Indonesia, Depok, Indonesia; 3 Department of Public Health, Faculty of Sport Science, Universitas Negeri Malang, Malang, East Java, Indonesia

**Keywords:** Tobacco use, male adolescents, smoking

## Abstract

**Objectives:**

The aim of the study was to examine the factors influencing cigarette consumption among male adolescents aged 15-24 years in Indonesia.

**Methods:**

This study used the Indonesia Demographics and Health Survey 2017, which included 8,488 male adolescents aged 15-24 years. The survey used multi-stage cluster sampling. Data were analyzed by using multivariable logistic regression adjusted for covariates and a complex survey design.

**Results:**

There is 70.4% of male adolescents who smoke any tobacco products daily or occasionally. The male adolescents who aged 20-24 years (p<0.001; AOR=2.26, 95%Cl=1.96-2.59), had low education level (p<0.001; AOR=5.90, 95%Cl=3.91-8.92), start smoking at 18-24 years (p-value<0.001; AOR=3.09, 95%Cl=2.25-4.23), had been influenced by friend/someone to smoke (p<0.001; AOR=5.60, 95%Cl=4.77-6.58), used the internet at least once a week (p<0.001; AOR=1.28, 95%Cl=1.11-1.49), did not read newspaper/magazine (p<0.001; AOR=1.55, 95%Cl=1.28-1.87) had a higher odds of current smoking.

**Conclusions:**

Factors of smoking tobacco, such as age, age at first smoking, low education, the influence of smoking, and access to information on the internet and newspapers/magazines, had a significant impact on the current tobacco of male adolescents. Our findings support the enforcement of health warnings and laws related to tobacco restrictions for adolescents.

## Introduction

Smoking is the leading cause of disease and death worldwide. Globally, tobacco use can cause health diseases such as coronary heart disease, stroke, and multiple types of cancer, including lung cancer 1. According to The 4^th^ edition of Tobacco Control Atlas, Indonesia is ranked as the third most cigarette user globally, with the most cigarette users in Southeast Asia 2. Basic Health Research in 2018 showed a trend of increasing the proportion of cigarette users in Indonesia annually. It was evidenced by the increasing prevalence of smoking in the population aged 10 to 18 years, namely by 1.9% from 2013 (7.2%) to 2018 (9.1%) 3.

Most people try smoking for the first time as adolescents 4. Based on data from The 4^th^ Edition of Tobacco Control Atlas (2018), smokers in Indonesia started smoking at the age of under 20 years. A study using the Global Tobacco and Youth Report (GYTS) Indonesia in 2019 also showed that males have has greater percentage of current smoking than females 6. Based on a report from the World Health Organization (WHO) on The Global Tobacco Epidemic in 2017, the prevalence of tobacco users at a young age in Indonesia currently reaches 12.7% 7.

The problem of cigarette consumption in Indonesia is very concerning. There were 469,000 children aged 10-14 years and 53.248.000 people aged 15 years and over who consumed tobacco daily in Indonesia in 2015^8^. The increase in the prevalence of smokers among adolescents is currently one of the focuses of the Indonesian Ministry of Health in preventing a growth in the majority of smoking in Indonesia 9. Indonesia's high number of smokers has resulted in many health and economic losses. The 4^th^ edition of Tobacco Control Atlas (2018) indicates that Indonesia has spent more than 569 trillion rupiahs on treating 28 diseases caused by smoking, such as chronic obstructive pulmonary disease, cardiovascular disease, and cancer 5. In addition to being bad for health, cigarette smoke harms the health of the passive smoker.

Adolescence is a time full of emotional turmoil and imbalance in trying to achieve an identity influenced by the surrounding environment 10. According to Mirnawati (2018), there are three factors that influence risky adolescent behavior. First, predisposing factors inherent in a person, such as knowledge, age, gender, and education. Second, enabling factors, such as the availability and affordability of health resources and housing. Finally, reinforcement factors such as family, society, and peers reinforce behavior. Therefore, it is important to examine factors that affect the occurrence of smoking in adolescents in Indonesia 11.

A previous study by Efendi et al. (2018) reported that most adolescents and young men in rural Indonesia smoke tobacco. This study indicates that age 20-24 years (OR=2.8), employment status (OR=2.24), and low level of education (OR=1.93) are significant factors in smoking among male adolescents in rural areas in Indonesia^13^. Additionally, smoking behaviour in rural and urban areas showed the difference results^14^. A study also stated that male adolescents had a higher risk of smoking behaviour than female adolescents 15.

A study by Smet et al. 16 examined smoking prevalence among male adolescents in Semarang. This study found that smking increased dramatically between the ages of 11 and 17, from 8.2% to 38.7%. The smoking behavior of best friends was the most powerful determinant of smoking, consistent across age groups. Best friends' attitudes towards tobacco and older brothers' smoking behavior were also important determinants of smoking. Also, other variables may be related to smoking 16.

Based on the Tobacco Atlas (2022), it was reported that global smoking prevalence has decreased from 22.75% in 2007 to 19.6% in 2019. Nevertheless, the total number of smokers remains a high challenge due to population growth. Furthermore, males were more likely to be current smokers compared to females in 2019 17. The aim of the study was to examine the factors influencing cigarette consumption among male adolescents aged 15-24 years in Indonesia. This research is expected to provide information about the factors that influence smoking in male adolescents and reduce or prevent increased smoking behavior and diseases caused by smoking. Additionally, this study presented the updated situation about current smokers and their risk factors in male adolescents using nationally representative data. It is important to understand whether there are new or evolving risk factors driving teenagers to smoke given the shift in smoking prevalence among this age group. Therefore, this study can serve as a foundation for developing initiatives to raise public awareness and implement tighter tobacco control laws, particularly in the adolescent population. As a sort of investment in the health of the future generation, efforts must be undertaken to safeguard kids against smoking and other tobacco-related risks.

## Methods

### Data Source

This study used secondary data from the 2017 Indonesia Demographic and Health Survey (2017 IDHS). The 2017 IDHS is part of the worldwide Demographic and Health Survey (DHS) program, which is conducted by the National Population and Family Planning Board (BKKBN) in collaboration with Indonesian Statistics (BPS) and the Indonesian Ministry of Health (MoH). The IDHS was a nationally representative dataset. The survey data is publicly available and accessible on the DHS program website (www.dhsprogram.com).

### Sample Size and Sampling

The 2017 IDHS sample covered 1.970 census blocks in urban and rural areas and was expected to obtain responses from 49.250 households. DHS determines the sample in two stages: first, using random cluster selection with Probability Proportional to Size (PPS) to identify the most significant clusters and randomly selecting households from an existing list of families; the second one is in each census block chosen, 25 ordinary households were selected with systematic sampling from the updated household listing. The detailed sampling strategy and data collection are given in the DHS reports.

The inclusion criteria in this study were male adolescents aged 15-24. From all sampled households, it was expected to obtain about 59.100 respondents of women aged 15-49, 24.625 respondents of never-married men aged 15-24, and 14.193 respondents of married men aged 15-54. Our study analyzed 8.488 male adolescents after excluding missing data.

### Data Collection

Prior to fieldwork, questionnaires, all instruments, and the procedures of the survey were tested to determine whether the questions were clear and could be understood by the respondents and to improve the implementation of the 2017 IDHS. The master instructors, field coordinators, national instructors, and interviewers were trained to transfer the same understanding of concepts and operational definitions of the variables collected in the survey. All completed questionnaires and control forms were returned to the BPS central office in Jakarta for data processing. The control forms were used to monitor the completeness and accuracy. The questionnaires were logged and edited, and all open-ended questions were coded. Responses were entered in the computer twice for verification, and they were corrected for computer-identified errors 18. The DHS dataset has specific values that are used throughout the recoded data file for certain responses. Two of them were very important, namely, not applicable and missing 19. Fieldwork took place from July 24–September 30, 2017 20.

## Study Variables

### Outcome Variables

The outcome variable was current smokers among male adolescents. The current smoker was an adolescent who, at the time of the survey, smoked any tobacco products daily or occasionally. The responses to the questions were every day, occasionally, and not at all. Respondents who answered every day or once in a while were categorized as current smokers, and respondents who answered not at all were classified as non-current smokers 19.

### Independent Variables

The independent variables were age, place of residence, education level, age at first smoking, influence to smoke, use of the internet, and access to information (television, radio, and newspapers/magazines). Age was grouped as 15-19 and 20-24 years. Rural and urban areas were the places of residence. Education level was grouped into primary, secondary (junior and senior high school), and higher (academy/D1/DII/DIII/diploma/university) categories. Age-first smoking was grouped as 4-10, 11-17, and 18-24 years. Ever asked or influenced a friend or someone to smoke was categorized as yes or no. The used internet was divided almost daily, at least once a week, and not used at all. Implementing partners of the DHS program are classified. Information access, newspapers/magazines, television, and radio into three categories (not used at all, less than once a week, at least once a week) 19.

### Statistical Analysis

We performed descriptive statistics to assess the distribution of participants. We presented them as frequencies (n) and proportions (%) of adolescents' current tobacco smoking risk estimated among the independent variables. We used the chi-square test to examine the relationship between variables and tobacco smoking. We also used univariable logistic regression and multivariable logistic regression to investigate the risk factors of tobacco smoking. Presented as a Crude Odd Ratio (COR) and Adjusted Odd Ratio (AOR) with 95% CI and p-value. Statistical significance was defined as a p-value of less than 0.05. STATA 14 was used to analyze the data.

## Results

### Characteristics of Respondent

Based on data collected from 8.488 respondents and evaluated, 70.4% of male adolescents at the time of the survey had smoked any tobacco products daily or occasionally. In contrast, 29.6% of male adolescents did not smoke at the time of the survey. According to age, 56.6% of male adolescents aged 15-19 and 43.4% of male adolescents aged 20-24 smoked during the survey. The results show that 56.1% of male adolescents live in urban areas, and 43.9% live in rural areas. As many as 79.3% of male adolescents had secondary education, and 7.17% had primary education. Based on the age of first smoking, it mostly occurred between the ages of 11 and 17 (83.77%). Based on access to internet use, 71.5% of male adolescents use the internet almost daily (Table 1).

**Table 1:** Characteristics of Respondent

Variables	n	%
**Current smoking**		
Yes	5.976	70.40
No	2.512	29.60

**Age of respondent (years)**		
15-19	4.804	56.60
20-24	3.684	43.40

**Residence**		
Urban	4.762	56.10
Rural	3.726	43.90

**Education**		
Primary	0.609	07.17
Secondary	6.708	79.03
Higher	1.171	13.79

**Age at first smoking (years)**		
4-10	0.661	07.78
11-17	7.110	83.77
18-24	0.717	08.45

**Used internet the last month**		
Almost every day	6.069	71.50
At least once a week	2.274	26.79
Not at all	0.145	01.71

**Ever asked/influenced a friend/someone to smoke**		
No	5.469	64.43
Yes	3.019	35.57

**Listens to radio**		
At least once a week	1.394	16.42
Less than once a week	2.824	33.27
Not at all	4.270	50.31

**Watches television**		
At least once a week	6.741	79.42
Less than once a week	1.626	19.15
Not at all	0.121	01.43

**Reads newspaper or magazine**		
At least once a week	1.302	15.34
Less than once a week	3.142	37.01
Not at all	4.044	47.65

According to ever asked/influenced a friend/someone to smoke, 64.43% of male adolescents admitted that they had never influenced a friend/someone to smoke, and 35.57% admitted that they had ever influenced a friend/someone to smoke. Regarding media exposure, we found that 50.3% of male adolescents do not listen to the radio, 16.42% listen to the radio at least once a week, and 33.27% listen less than once a week.

Concerning television use, 79.42% of male adolescents watch television at least once a week, 19.5% watch television less than once a week, and 1.43% do not. Related to reading newspapers/magazines, most male adolescents do not read newspapers/ magazines (47.05%). This number is quite higher than at least once a week (15.34%) and 37.01% less than once a week (Table 1). The distribution of factors based on the influence of smoking is listed in Table 2.

**Table 2:** Distribution Factor of Male Adolescents Smoking

Current Smoking								

Variables	n		Yes		No			p-value

		n	%	95%CI	n	%	95%CI	
**Age of respondent (years)**								
15-19	4804	3097	64.46	[62.56-66.33]	1.707	35.54	[33.67-37.44]	≤0.001
20-24	3684	2879	78.15	[76.41-79.80]	6.290	21.85	[20.20-23.59]	

**Residence**								
Urban	4762	3263	68.52	[66.66-70.32]	1.499	31.48	[29.68-33.34]	0.003
Rural	3726	2713	72.82	[70.61-74.91]	1.013	27.18	[25.09-29.39]	

**Education**								
Primary	609	543	89.09	[85.31-91.98]	0.066	10.91	[08.02-14.69]	
Secondary	6708	4743	70.7	[69.13-72.22]	1.965	29.30	[27.78-30.87]	≤0.001
Higher	1171	691	59.02	[55.35-62.58]	0.480	40.98	[37.42-44.65]	

**Age at first smoking (years)**								
4-10	661	367	55.56	[51.17-59.87]	0.294	44.44	[40.13-48.83]	
11-17	7110	5034	70.8	[69.24-72.31]	2.076	29.20	[27.69-30.76]	≤0.001
18-24	717	575	80.17	[76.36-83.49]	0.142	19.83	[16.51-23.64]	

**Often used the internet last month**								
Almost every day	6069	4149	68.37	[66.66-70.02]	1.920	31.63	[29.98-33.34]	
At least once a week	2274	1717	75.51	[73.37-77.52]	0.557	24.49	[22.48-26.63]	≤0.001
Not at all	145	110	75.71	[66.49-83.04]	0.035	24.29	[16.96-33.51]	

**Ever asked/influenced a friend/someone to smoke**								
Yes	3019	2694	89.22	[87.77-90.52]	0.325	10.78	[9.48-12.23]	≤0.001
No	5469	3282	60.02	[58.14-61.86]	2.187	39.98	[38.14-41.86]	

**Listens to radio**								
At least once a week	1394	997	71.51	[68.48-74.35]	0.397	28.49	[25.65-31.52]	
Less than once a week	2824	1924	68.13	[65.91-70.28]	0.900	31.87	[29.72-34.09]	0.031
Not at all	4270	3055	71.55	[69.62-73.40]	1.215	28.45	[26.60-30.38]	

**Watches television**								
At least once a week	6741	4683	69.47	[67.91-70.99]	2.058	30.53	[29.01-32.09]	
Less than once a week	1626	1210	74.43	[71.37-77.26]	0.416	25.57	[22.74-28.63]	0.021
Not at all	121	83	68.29	[54.90-79.20]	0.038	31.71	[20.80-45.10]	

**Reads newspaper or magazine**								
At least once a week	1302	827	63.55	[60.07-66.89]	0.475	36.45	[33.11-39.93]	
Less than once a week	3142	2113	67.25	[65.10-69.33]	1.029	32.75	[30.67-34.90]	≤0.001
Not at all	4044	3035	75.06	[73.14-76.89]	1.009	24.94	[23.11-26.86]	

### Factor Associated with Smoking behavior

According to the result of multivariable analysis, male adolescents aged 20-24 years were two times (AOR 2.26; 95%Cl 1.96 to 2.59) more likely to smoke tobacco compared with male adolescents aged 15-19 years in Indonesia. Male adolescents with primary and secondary education had two (AOR 2.20; 95%CI 1.81 to 2.67) and almost six-time (AOR 5.90; 95%CI 3.91 to 8.92) higher odds ratio, respectively, of having self-reporting tobacco smoking smoke tobacco than those with higher education. Our results also showed that male adolescents who started smoking at 18-24 years old were three times (AOR 3.09; 95%Cl 2.25 to 4.23) more likely to smoke tobacco than those who began smoking 4-10 years and male adolescents who started smoking at 11-17 years old were two times (AOR=2.04; 95%CI 1.67 to 2.50) more likely to smoke tobacco than those who began smoking 4-10 years.

Male adolescents who ever asked/influenced a friend/someone to smoke were five times higher (AOR 5.60; 95%Cl 4.77 to 6.58) than those who never requested/influenced a friend/someone to smoke. According to internet access, this study found that male adolescents who used the internet at least once a week had a one times (AOR 1.28; 95%Cl 1.11 to 1.49) risk of smoking than those every day according to access information such as newspapers/ magazines, a male adolescent who do not access newspaper/magazine (AOR 1.55; 95%Cl 1.28 to 1.87) had one time to smoking tobacco. Place of residence, watching television and radio, did have an insignificant relationship with current smoking.

## Discussion

Humans behave consciously and consider all available information. In the Theory of Planned Behavior, behavior is not only determined by attitudes and subjective norms alone. Background factors, such as personal components, social components (age, gender, ethnicity, socio-economic status, education, religion), and information components (experience, knowledge, media exposure), influence individual attitudes and behavior towards something 21. This theory has previously been used for research on smoking in numerous nations as well as for predicting smoking behavior 22,23.

Our study showed that 70.4% of male adolescents smoke tobacco, and 29.6% are non-smokers. Our study found that adolescents aged 20-24 have a high smoking risk. These findings have the same results as previous studies by Efendi et al. 13 stated that a significant factor related to smoking in adolescents is the age of 20–24 years, with a two times greater risk of smoking. In addition, an analysis of clinical data from the Victorian Aboriginal Community Controlled Health Organization (VACCHO) member service stated that smoking prevalence was higher at 20-24 years 24. Many factors influence adolescents to smoke a lot at 20-24. Previous research stated that at that age, adolescents experience a period of development marked by physical, psychological, social/cultural, and cognitive changes. Besides that, adolescents begin to search for identity by exploring the environment and friends, and they greatly influence and express it by taking risks 25,26.

Previous research in Syria also showed that smoking in urban areas (52.2%) was higher than in rural areas (50.4%) 27. Another research claim was urban areas are strongly associated with smoking 28. In contrast to previous research, our research yielded insignificant results on the place of residences smoking has become a behavior that is rooted in the structure of urban and rural communities. After all, it has become a habit and culture carried out continuously 29,30. In society, smoking is a form of representation of manhood, and this paradigm gives rise to the interpretation that smoking is absolute behavior for men 31. This culture formed the concept that “every man must smoke, and if you don't smoke, you cannot be called a man.” The term says you are not a man if you don't smoke 29. Another factor is related to the ease of access to the internet both in urban and rural areas, which display an increasingly diverse variety of cigarette advertisements. Even cigarette advertisements show various flavors (cigarettes) themselves 32,33.

Based on educational status, male adolescents who were not formally educated either can read/write or not significantly increased the likelihood of smoking. Some studies also found that higher education students have a lower risk of smoking 12,34. Lawrence 35 confirms that more educated individuals will see results from their actions, encouraging and enabling healthier behaviors. Besides education, another factor that influences the occurrence of smoking is the age at first smoking. This study states that male adolescents with a high risk of first smoking are 18-24 years old. These results align with previous research, which said that 18-24 years old are prone to depression and anxiety, so they are strong predictors of smoking trials and switching to smoking habits among adolescents 36.

Adolescence is a transitional period that is vulnerable to biological and psychosocial development 37, so at that age, the friendship environment greatly influences adolescent behavior 38. Friendship is the most significant influence in influencing someone to start smoking, besides that the impact of differences in culture, society, values, and social norms, as well as changes in roles, responsibilities, relationships, and expectations, can influence adolescents to start smoking 39,40. Regarding the influence of friends to smoke, this study shows that individuals who influence others to smoke had a five times greater risk of making others smoke. The statement is explained according to the principle of homophily, that individuals prefer to associate with those with the same characteristics, beliefs, and behaviors as them 41,42.

Adolescents who are well-informed about health issues are more likely to be able to filter through the available information and decide which of it should be taken into account for everyday behavior. They can access a variety of information sources, including the internet, radio, television, newspapers, and magazines, to learn about health-related topics, including smoking. The meta-analysis study showed that the inadequate health literacy group had a higher odds of being a current smoker than the adequate health literacy group (OR 1.49; 95% CI: 1.25–1.79) 43. People with low health literacy could be less aware of the harmful consequences of smoking on their health as well as less able to comprehend and utilize smoking cessation treatments. Based on The Health Literacy Skills framework, health literacy highlights how health literacy operates at the individual level and has a relationship to health-related outcomes 44.

Our study found that male adolescents who use the internet at least once a week have a one-time higher risk of smoking. Another previous research claims that someone who uses the internet excessively will increase smoking behavior because excessive internet use will reduce health conditions such as stress, negative emotions, and poor impulse control, which will trigger someone to smoke. They vented their feelings on cigarettes 45,46. Another factor that influences current smoking among male adolescents is information access. We found that accessing information such as the internet and reading newspapers/magazines negatively correlated with tobacco but were much weaker in magnitude. This finding is supported by Getachew et al. 33 found that internet access was one time more likely to cause someone to smoke, probably due to exposure to advertising. Another research reported that tobacco advertisements through print media (magazines/ newspapers), letters, and television significantly affect tobacco use in adolescents 47.

Our study found that access to information, such as listening to the radio and watching television, has no significant relationship with tobacco smoking. Previous research by Achia 48 stated that watching television had no significant association with tobacco use. Another study also claims that listening to the radio has no significant association with tobacco use 49. Radio and television are currently being threatened due to new technological communication media. Radio and television are considered traditional communication tools because their presence has been replaced by gadgets, especially now that teenagers access the internet more via mobile phones than by watching television and listening to the radio 50,51.

DHS data uses a cross-sectional design, and deducing the causes and consequences of their occurrence is difficult. Factors influencing the incidence of smoking in adolescents in DHS were determined using self-survey or recall methods, causing information bias because respondents were asked to remember the age of the first time they smoked. On the other hand, this study has a reasonably high response rate and low bias measurement. All procedures and instruments are validated. Before the survey, field workers were also educated to ensure they consistently understood the operational concept of variables.

There is a need to be cooperation between the Ministry of Health, the Ministry of Education and Culture, and the Ministry of Communication and Informatics, namely by having a prevention program through health education by peer educators as an effort to prevent the dangers of smoking to increase the knowledge and attitudes of peer groups. In addition, it is necessary to simplify supervision regarding cigarette advertisements through print, electronic, and social media among adolescents in Indonesia. Media marketing interests millennials because content needs to be distributed through social networks or adolescent interactions. The popularity of social media allows for increasing the visibility of media campaigns, extends the reach of messages, and can increase the effectiveness of health promotion; this is also supported by public health organizations which are increasingly turning to social media to spread health campaigns, this was chosen because it can access a wide audience “hidden” or at risk, such as adolescent. The government must strengthen and optimize the implementation of Government Regulation No. 109 of 2012, Article 39, which prohibits anyone from broadcasting and depicting in the form of images or photos, showing, displaying, or revealing individuals smoking, showing cigarettes, cigarette smoke, cigarette packaging, or anything related to tobacco products, as well as any form of tobacco product information in print media, broadcasting media, and information technology media related to commercial/advertising activities or promoting smoking. Law Number 32 of 2002, Article 46, on broadcasting also prohibits the promotion of cigarettes by showcasing their appearance. Furthermore, in Government Regulation Number 50 of 2005, cigarette advertisements on radio and television are only broadcast from 21:30 to 05:00 local time.

## Conclusion

Male adolescents aged 20–24 years who had low education, were over ten years old when they first smoked, were influenced by friends or someone to smoke, used the internet at least once a week, and did not read newspapers or magazines had a higher risk of being current smokers. Our findings support the enforcement of health warnings and laws related to tobacco restrictions for adolescents. In this regard, we suggest involving social media for health promotion. Leveraging cultural elements in social media, such as popular content creators (influencers) and messages (memes), is a promising strategy to inform strategies for spreading education and health campaigns effectively.

## Figures and Tables

**Table 3:** Association between Independent Variables and Male Smoking Behavior

Variables	Bivariable			Multivariable		

	COR	95%Cl	p-value	AOR	95%Cl	p-value
**Age of respondent (years)**						
15-19	Ref			Ref		
20-24	1.97	1.75 - 2.22	≤0.001	2.26	1.96 - 2.59	≤0.001

**Residence**						
Urban	Ref			Ref		
Rural	1.23	1.07 - 1.41	0.003	1.11	0.96 - 1.30	0.163

**Education**						
Primary	0.29	0.20 - 0.41	≤0.001	5.90	3.91 - 8.92	≤0.001
Secondary	0.17	0.12 - 0.25	≤0.001	2.20	1.81 - 2.67	≤0.001
Higher	Ref			Ref		

**Age first smoking (years)**						
4-10	Ref			Ref		
11-17	1.93	1.61 - 2.33	≤0.001	2.04	1.67 - 2.50	≤0.001
18-24	3.23	2.44 - 4.26	≤0.001	3.09	2.25 - 4.23	≤0.001

**Ever asked/influenced a friend/someone to smoke**						
Yes	5.51	4.71 - 6.44	≤0.001	5.60	4.77 - 6.58	≤0.001
No	Ref			Ref		

**Often used the internet last month**						
Almost every day	Ref			Ref		
At least once a week	1.42	1.25 - 1.62	≤0.001	1.28	1.11 - 1.49	≤0.001
Not at all	1.44	0.91 - 2.27	0.114	0.92	0.59 - 1.44	0.712

**Listens to radio**						
At least once a week	Ref			Ref		
Less than once a week	0.85	0.72 - 1.00	0.061	0.86	0.72 - 1.04	0.118
Not at all	1.00	0.85 - 1.18	0.981	0.97	0.81 - 1.16	0.745

**Watches television**						
At least once a week	Ref			Ref		
Less than once a week	1.27	1.08 - 1.51	0.004	1.14	0.96 - 1.36	0.142
Not at all	0.94	0.53 - 1.67	0.849	0.80	0.43 - 1.49	0.478

**Reads newspaper or magazine**						
At least once a week	Ref			Ref		
Less than once a week	1.17	0.99 - 1.39	0.055	1.17	0.97 - 1.40	0.103

Not at all	1.72	1.44 - 2.06	≤0.001	1.55	1.28 - 1.87	≤0.001
